# Evaluation of psychometric properties of needs assessment tools in cancer patients: A systematic literature review

**DOI:** 10.1371/journal.pone.0210242

**Published:** 2019-01-08

**Authors:** Lang Tian, Xiaoyi Cao, Xielin Feng

**Affiliations:** 1 Department of hepatobiliary surgery, Sichuan Cancer Hospital, Chengdu, Sichuan province, People’s Republic of China; 2 Hemodialysis Center, Department of Nephrology, West China Hospital, Sichuan University, Chengdu, Sichuan province, People’s Republic of China; Netherlands Comprehensive Cancer Organisation (IKNL), NETHERLANDS

## Abstract

**Background:**

Although a wide range of needs assessment tools for cancer patients have been developed, no standardized and commonly accepted instruments were recommended to use in clinical care. This systematic review was conducted to assess the quality of psychometric properties of needs assessment tools among cancer patients in order to help oncology healthcare professionals select the most appropriate needs assessment tools in routine clinical practice.

**Methods:**

Searches were conducted in the electronic databases of PUBMED from 1966, CINAHL from 1960, EMBASE from 1980 and PsychINFO from 1967 as well as additional sources. The quality of psychometric properties of the recruited needs assessment tools was evaluated using the agreed quality criteria for measurement properties of health status questionnaires.

**Results:**

Thirty-seven studies which evaluated the psychometric properties of 20 needs assessment tools were identified. Internal consistency was tested in 32 studies with 9 studies indicating negative rating and 4 studies intermediate rating. Less than half of the studies (13 studies) assessed test-retest reliability, and only 4 studies reported positive rating. Content validity was the most tested psychometric property appraised in 33 studies and indicated positive rating in all the evaluated studies. Structural validity was adequately evaluated in 28 studies with 23 studies reporting intermediate rating. More than half of the studies (29 studies) tested hypothesis testing and 13 studies were rated positive. Cross-cultural validity results were obtained in 13 studies with 7 studies showing negative rating. No data was available on measurement error and criterion validity. Only one study appraised responsiveness and showed intermediate rating. The Supportive Care Needs Survey-Short Form (SCNS-SF) is the most widely used instrument for needs assessment in cancer patients. It had strong evidence for internal consistency, content validity, structural validity and hypothesis testing, and moderate evidence for reliability and cross-cultural validity. Cancer Survivors’ Unmet Needs Measure (CaSUN) reported strong or moderate evidence for internal consistency, reliability, content and structural validity, and hypothesis testing. Furthermore, Supportive Cancer Care Needs Assessment Tool for Indigenous People (SCNAT-IP) had strong evidence for content validity, and moderate evidence for internal consistency, structural validity and hypothesis testing.

**Conclusions:**

Despite several needs assessment tools exist to assess care needs in cancer patients, further improvement of already existing and promising instruments is recommended.

## Introduction

Cancer is one of the leading causes of morbidity and mortality around the whole world, with approximately 14.1 million new cancer cases, 8.2 million cancer deaths, and 32.6 million people living with cancer in 2012. [1] In 2016, cancer is the second leading cause of non-communicable disease (NCD) deaths (9.0 million or 22% of all NCD deaths) globally. [2] Moreover, in 2018, there are an estimated 3.91 million new cases of cancer and 1.93 million deaths from cancer in Europe where a total population that comprises 9.0% of the world’s population. [3] Throughout their disease and treatment trajectories, several cancer patients suffer from a wide range of disease- and treatment-related side effects and symptom distress, which can impair their health-related quality of life (HRQOL) and make it difficult for them to get through treatment. [4,5] In addition to prolonging life, the maintenance and improvement of HRQOL is a critically important goal of integrated and patient-centered cancer care. [6] However, patient-centered care cannot be fully provided without a better assessment and understanding of patient care needs and the variables that affect them. [7] Meanwhile, several previous studies have demonstrated that, unmet care needs were significant contributors to poor HRQOL among cancer patients. [8–10] Therefore, it is crucial for oncology healthcare professionals to identify and manage the unmet care needs of cancer patients effectively in order to enhance and maintain their HRQOL.

A rigorous and systematic needs assessment is the crucial first step in integrated and patient-centered cancer care. [7] Needs assessment addresses a comprehensive appraisal of care needs of the individuals (e.g., physical, psychological, social, spiritual, financial, information and health care needs), and can help identifying whether or not the individuals want help and provide insights into the magnitude of that need. [11] Needs assessment in cancer patients is an ongoing process which is recommended to be carried out from pre-diagnosis to cure, progressing disease or death into bereavement. [7] Accurate and effective needs assessment can assist in prioritizing care needs, allocating resources to the areas and individuals that need them most, developing more appropriate and cost effective patient care strategies, and improving HRQOL eventually. [12]

Moreover, regarding the needs assessment tools in cancer patients, a previous literature review conducted in 2007 has identified 15 tools which have been developed from 1984 to 2004, and has appraised and compared their validity, reliability, responsiveness and feasibility. [7] Nevertheless, the findings indicated that none were found to meet all the acceptable criteria for measurement properties, and none were recommended to use in clinical care. Furthermore, some instruments recruited in the literature review such as Cancer Care Monitor (CCM) and Symptom and Concern Checklist (SCC) have primarily focused on assessing the prevalence and severity of symptoms, but not on the evaluation of cancer care needs. [7] Recently, several new cancer-specific needs assessment tools have been developed, and the most commonly used instruments are composed of Supportive Care Needs Survey-Short Form (SCNS-SF), Cancer Survivors’ Unmet Needs Measure (CaSUN), Survivors Unmet Needs Survey (SUNS), and Needs Based Biopsychosocial Distress Instrument for Cancer Patients (CANDI). [13–16] However, their psychometric properties have not been systematically reviewed and compared.

In addition, although a variety of cancer-specific care needs assessment tools have been developed in recent years, there is still a lack of standardized and commonly accepted tools for a comprehensive evaluation of care needs among cancer patients in routine clinical practice. That may be attributed to the fact that there is not a comprehensive and systematic appraisal of measurement properties of cancer-specific needs assessment tools, which the agreed quality criteria for measurement properties of health status questionnaires have recommended recently. [17] A systematic review of psychometric properties of the recruited instruments comprising their validity, reliability and responsiveness should be carried out to rate their quality. Therefore, the purpose of the study was to perform a systematic review on the quality of psychometric properties of needs assessment tools among cancer patients in order to make recommendations on the most appropriate instruments for care needs assessment for cancer patients through collecting evidence from previous studies.

## Methods

### Inclusion and exclusion criteria

The studies that met the following criteria were eligible in the systematic review: (1) recruited adults with cancer as the samples; (2) originally aimed to develop instruments to measure comprehensive care needs specifically for multiple cancer patients (cancer-specific need assessment tools), or assess cross-cultural adaptation of these tools; (3) reported the psychometric properties of these instruments; and (4) have been published in English language. The studies meeting the following criteria were excluded: (1) aimed to develop tools originally to test care needs in single site cancer patients such as breast cancer, prostate cancer, or head and neck cancer; (2) evaluated the psychometric properties of the instruments originally developed to assess care needs in other chronic illnesses; (3) only assessed unidimensional care needs such as physical, psychological, social or communication needs; (4) evaluated experienced problems in health status (e.g., the prevalence and severity of symptom distress, and HRQOL) and the quality of care; and (5) interventional study, qualitative study, cross-sectional descriptive study, discussion paper, literature review, and guideline.

### Search strategy

The search for articles was conducted in the electronic databases of PUBMED from 1966, CINAHL from 1960, EMBASE from 1980, and PsychINFO from 1967 to 31^st^ August 2018. A combination of Medical Subject Headings and keywords was used in the systematic literature searching procedure: (neoplasm* or cancer* or carcinoma* or tumor*or oncology* or malignan* or lymphoma or melanoma or leukemia or sarcoma) AND (need*) AND (evaluation* or assessment* or psychometric* or measure* or propert* or develop* or reliab* or valid* or responsive*or method* or tool* or instrument* or scale* or survey* or questionnaire* or instrument* or version*). Grey literatures were extracted from Google scholar. The search strategy in each database was presented in S1 Appendix.

The literature screening procedure was performed by two independent reviewers (TL and CXY). First, article titles and abstracts were screened for eligibility by one reviewer (TL). Then, a second reviewer (CXY) checked and verified the screening process. Articles that did not meet the inclusion criteria were excluded based on their titles or abstracts firstly. When the relevance of an article was not clear according to the abstracts, both reviewers (TL and CXY) checked the final inclusion depending on retrieving full-text articles. Discrepancies or inconsistency were resolved by consensus or discussing with a third reviewer (FXL).

### Data extraction and synthesis

Two reviewers (TL and CXY) extracted information from articles that met the inclusion criteria using a pre-designed structured data extraction form. The specific data information comprised name of the instruments, language versions, target population and settings, number of items and domains, response format and completion time, and instrument reliability (internal consistency and test-retest reliability), validity (content validity, structural validity, convergent validity, discriminant validity and cross-cultural validity) and responsiveness. If there were missing data, the authors of included studies were contacted for further details. Discrepancies or inconsistency were also resolved by consensus or discussing with a third reviewer (FXL).

### Evaluation of the methodological quality of each study

The methodological quality of studies on the psychometric properties of needs assessment tools was evaluated by the Consensus-Based Standards for the Selection of Health Measurement Instruments (COSMIN) checklist. [18,19] Based on the taxonomy and definitions of the COSMIN checklist, the methodological quality of studies is assessed by 9 measurement properties including internal consistency, test-retest reliability, measurement error, content validity, criterion validity, structural validity, hypothesis testing, cross-cultural validity and responsiveness. [20] The evaluation of the methodological quality of studies of each psychometric property comprises 5–18 items, and each item is rated on a four-point rating scale: poor, fair, good and excellent. The total score of methodological quality of each study is determined by a measurement property, and a methodological quality score of each measurement property can be obtained by taking the lowest score of any item in each measurement property. [20] The methodological quality on criterion validity was not examined in the review, as no gold standard for cancer-specific needs assessment tools can be found. Quality on measurement error was also not assessed, because none of the studies tested it.

### Evaluation of the quality of psychometric properties

The updated quality criteria for measurement properties of health status questionnaires originally developed by Terwee et al. (2007) was used to appraise the quality of psychometric properties of cancer-specific needs assessment tools in the systematic review (S1 Table). [21] It is composed of 9 measurement properties: 3 reliability indexes (internal consistency, test-retest reliability and measurement error), 5 validity indexes (content validity, structural validity, criterion validity, hypothesis testing and cross-cultural validity), and responsiveness with a four-point rating scale: positive (+), indeterminate (?), negative (-), and no data available (0). [21] For instance, a positive rating (+) is given to test-retest reliability if Intraclass Correlation Coefficient (ICC) or weighted Kappa is ≥ 0.70; an indeterminate rating (?) is given with no ICC or weighted Kappa calculated; a negative rating (-) is given with ICC or weighted Kappa < 0.70; and a zero rating (0) is given if no data can be available. The quality of criterion validity was not appraised in the review, as no gold standard for cancer-specific needs assessment tools was found and no recruited instruments reported their criterion validity. Meanwhile, measurement error was not tested, as none of the studies reported it. Furthermore, an evidence synthesis across studies was carried out for psychometric properties. The overall level of evidence for each tool was provided by one or more studies, according to their methodological quality (S2 Table). [21]

## Results

### Study selection process

**Fig 1:**
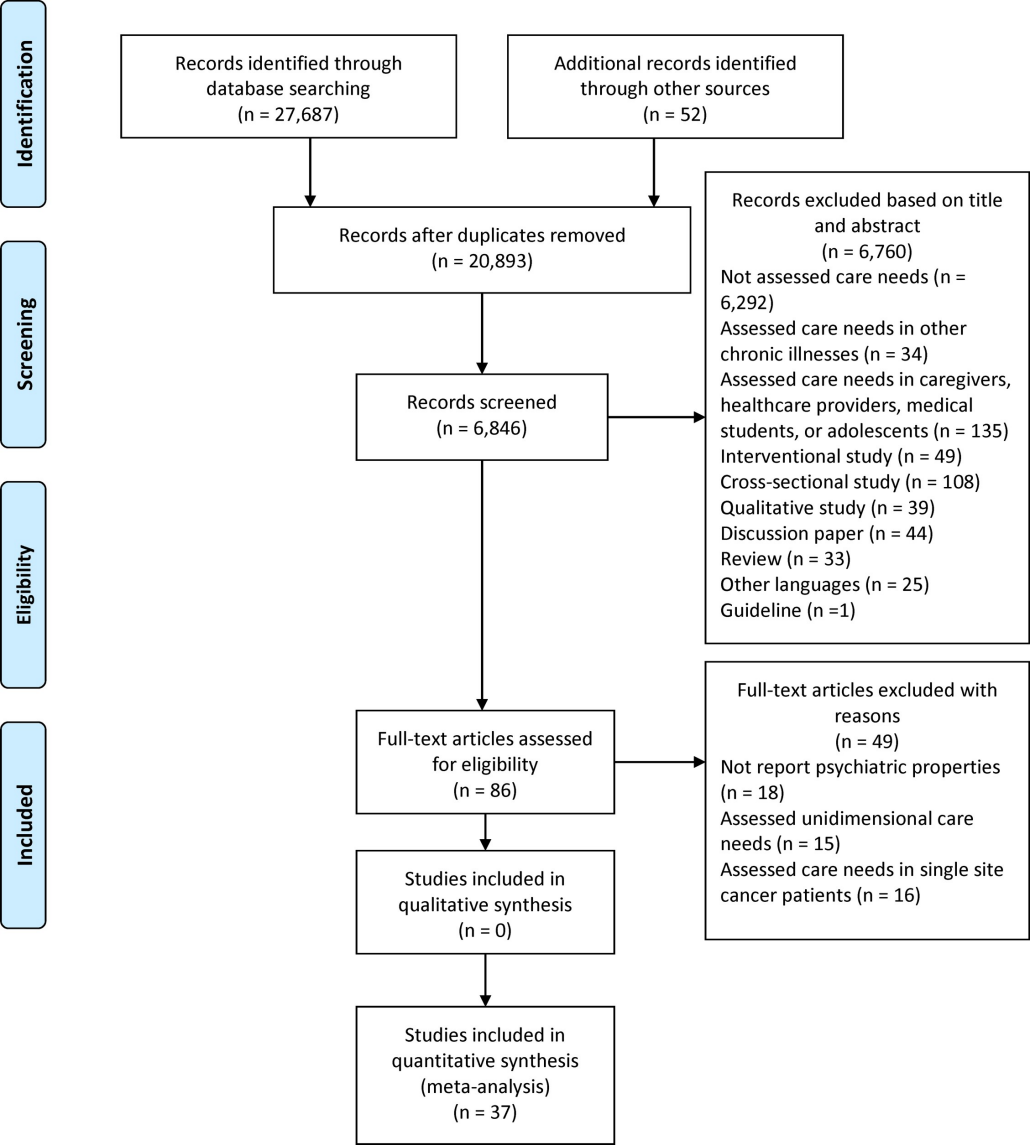
Selection of studies flowchart.

Our search of the electronic databases has identified 27,739 possible relevant articles primarily. After the literature screening procedure, 37 studies which have evaluated the psychometric properties of 20 needs assessment tools in cancer patients were identified in the summary of evidence (Fig 1).

### Characteristics of the study population

Among the 37 studies identified, 28 studies recruited multiple cancer patients as samples (e.g., breast cancer, prostate cancer, lung cancer, colorectal cancer, gastrointestinal cancer and other cancer), [13–16,22–45] and another 8 studies recruited a single site tumor sample for further verifying the psychometric properties of these instruments originally designed for multiple cancer patients, respectively. [46–53] Only one study did not report the included samples. [54] Of those, 4 studies recruited breast cancer patients, [46–48,51] and 4 other studies recruited subjects with head and neck cancer, prostate cancer, hematological cancer, and lung cancer, respectively. [49,50,52,53] Moreover, 8 studies were carried out in inpatient setting, [29,30,35,44–46,52,53] 17 in outpatient setting, [14–16,23,25,28,32–36,39,40,47,48,51,52] 8 in inpatient and outpatient settings, [22,24,27,38,41,42,45,46] and 4 studies did not report where the study was conducted. [13,29,31,54] (Table 1)

**Table 1:** Characteristics of the study populations and the needs assessment tools in cancer patients.

Instruments	Language	Country	Year	Target population	Setting	Time	Number of items	Number of domains	Response options
SCNS-SF	English [13]	Australia	2009	888 patients with breast, colorectal, prostate, lung and other cancer	†	10 min	34	5 domains: psychological, health system and information, physical and daily living, patient care, and sexuality	Five-point scale (1 = no need/not applicable, 2 = no need/satisfied, 3 = low need, 4 = moderate need, 5 = high need)
	French [46]	France	2012	384 breast cancer patients	Inpatient and outpatient	†	34	Same domains as [13]	Same response format as [13]
	German [22]	Germany	2012	1047 patients with breast, prostate, gastrointestinal tract and other cancer	Inpatient and outpatient	†	34	Same domains as [13]	Same response format as [13]
	Japanese [47]	Japan	2009	408 women with breast cancer	Outpatient	†	34	Same domains as [13]	Same response format as [13]
	Traditional Chinese [23]	Hong Kong and Taiwan	2013	360 colorectal cancer patients (Hong Kong) and 263 cancer patients (Taiwan)	Outpatient	†	34	Same domains as [13]	Same response format as [13]
	Mandarin [24]	China	2017	861 patients with gastrointestinal tract, liver, breast and other cancer	Inpatient and outpatient	†	33	Same domains as [13]	Same response format as [13]
	Mandarin and Cantonese [48]	Hong Kong	2011	348 women with breast caner	Outpatient	10 min	33	4 domains: health system, information and patient care, psychological, physical and daily living, and sexuality	Same response format as [13]
	Spanish [25]	Mexico	2015	825 patients with gastrointestinal tract, breast, genital and other cancer	Outpatient	†	33	Same domains as [13]	Same response format as [13]
	Dutch [49]	Netherlands	2016	201 patients with head and neck cancer	Inpatient	†	34	Same domains as [27]	Same response format as [13]
	English [50]	Australia	2012	332 men with prostate cancer in radiotherapy	Inpatient	†	34	Same domains as [13]	Four-point scale (1 = no need, 4 = high need)
SCNS-ST9	English [26]	Australia	2012	1458 patients with breast, lung, prostate, bowel/colon/rectum, and other cancer	Outpatient	†	9	5 domains: psychological, health system and information, physical and daily living, patient care and support, and sexuality	Same response format as [13]
SCNAT-IP	English [27]	Australia	2015	248 patients with breast, respiratory and intrathoractic, lymphoid, and other cancer	Inpatient and outpatient	†	26	4 domains: physical and psychological, hospital care, information and communication, and practical and cultural needs	Same response format as [13]
CANDI	English [16]	United States	2012	100 patients with breast, chronic lymphocytic leukemia, colon/rectal and other cancer	Outpatient	8 min	39	7 domains: depression, anxiety, emotion, social, healthcare, practical, and physical	Five-point scale (1 = not a problem, 5 = very severe problem) Additional choices are Prefer not to answer or Do not know
	Turkish [28]	Turkey	2016	172 patients with breast, colon, gastric, lung, and other cancer	Outpatient	†	39	3 domains: emotional, physical and social	†
CARES-SF	English [31]	United States	1991	3 samples, 479 cancer patients (sample 1), 1047 cancer patients (sample 2) and 114 newly diagnosed breast cancer patients (sample 3)	†	†	59	6 summary scales: global CARES, physical, medical, marital, psychosocial, and sexual	Five-point scale (0-not at all, 4-very much) Patients need to answer (Do you want help) (yes/no)
CARES	English [29]	United States	1988	2 samples, 479 cancer patients (sample 1), 1047 cancer patients (sample 2)	†	18 min	139	5 summary scales: physical, medical, marital, psychosocial, and sexual	Same response format as [36]
	Flemish [30]	Belgium	2016	176 non-palliative patients with breast, colorectal, and other cancer	Inpatient	31 min	130	5 summary scales: physical, medical, marital, psychosocial and sexual	Same response format as [36]
CaSUN	English [14]	Australia	2007	353 patients with breast, prostate, colorectal and other cancer	Outpatient	10 min	28	5 domains: existential survivorship, comprehensive care, information, QOL, and relationships	Three-point scale (met need, unmet need, total need)
	Dutch [32]	Netherland	2017	722 patients with breast, colon, and other cancer	Outpatient	†	37	6 domains: existential survivorship, comprehensive care, information, QOL, relationships, and lifestyle and return to work	Same response format as [14]
	Chinese [51]	Taiwan	2018	150 breast cancer patients (sample 1), 162 breast cancer patients (sample 2)	Outpatient	†	20	4 factors: information, physical /psychological, medical care, and communication needs	Same response format as [14]
SUNS	English [15]	Canada	2011	550 patients with breast, prostate, colorectal, lung, and other cancer	Outpatient	†	89	5 subscales: information, financial concerns, access and continuity of care, relationships, and emotional health	Five-point scale (0 = no unmet need, 4 = very high unmet need)
	English [52]	Australia	2014	715 hematological cancer survivors	Outpatient	†	89	5 subscales: information, financial concerns, access and continuity of care, relationships and emotional health	Same response format as [15]
SUNS-SF	English [33]	Canada	2014	1589 patients with breast, prostate, colorectal, lung, and other cancer	Outpatient	†	30	4 subscales: information, financial concerns, access and continuity of care, and relationships and emotional health	Same response format as [15]
SPARC	English [54]	United Kingdom	2004	†	†	45 min	45	7 domains: communication and information, physical, psychological, religious and spiritual, independence and activity, family and social, treatment and personal issues	Items, help/information/ contact with professionals (yes/no) Other items, four-point scale (0 = not at all, 4 = very much)
	Polish [34]	Poland	2012	58 advanced cancer patients with lung, colon, prostate, breast, and other locations	Outpatient	†	39	6 subscales: family and social, psychological, physical, independence and activity, treatment, and religious and spiritual	Same response format as [41]
NA-ACP	English [35]	Australia	2005	246 advanced, incurable patients with breast, bowel/colon, lung, lymph code, and other cancer	Outpatient	76 min	95	7 domains: medical communication/ information, psychological/emotional, daily living, financial, symptom, spiritual, and social	Same response format as [13]
NA-ALCP	English [53]	Australia	2012	108 advanced lung cancer patients	Inpatient	†	38	7 domains: medical communication, psychological/emotional, daily living, financial, symptom, spiritual/existential, and social	Same response format as [30]
SPEED	English [36]	United States	2011	53 active patients with breast, colon, lung, lymphoma and other cancer	Inpatient	†	120	5 domains: physical, spiritual, social, therapeutic and psychological	Likert 0–10 scale (0 = not at all and 10 = a great deal)
3LNQ	Danish [37]	Denmark	2011	74 advanced patients with gastrointestinal tract, breast, and other cancer	Inpatient	†	16	3 domains: problem intensity, problem burden, and felt need	Problem burden (not at all to very much) Feel need (does not have a problem-no need; has a problem but does not want help-no need; met need; unmet need; partially unmet need
CNAT	Korean [38]	South Korea	2011	2661 patients with stomach, lung, liver, colon/rectum, breast, cervix and other cancer	Inpatient and outpatient	†	51	7 domains: healthcare staff, physical symptoms, psychological problems, information, social/religious/spiritual support, practical support, and hospital facilities and services	Same response format as [30]
CNQ-SF	English [39]	Australia	2004	450 patients with lung, head and neck, gynaecological, and other cancer	Outpatient	†	32	5 domains: psychological, health information, physical and daily living, patient care and support, and interpersonal communication	Same response format as [13]
PNPC	Dutch [40]	Netherland	2004	64 cancer patients with distant metastasis (breast, colon/rectum, etc)	Outpatient	†	138	Both problem aspect and need for care aspect, ADL & IADL, physical symptoms, role activities, financial/administrative, social, psychological, spiritual, autonomy, problems in consultations, overriding problems in the quality of care Need for care aspect, concerning the general practitioners, concerning the specialist, informational needs	The PNPC asks 2 questions at each item, 1. Is this (item) a problem? Yes/Somewhat/No 2. Do you want (professional) attention for this (item)? Yes/As much as now/No
ISQ	Greek [41]	Greece	2016	109 patients with gastrointestinal, breast, lung, and other cancer	Inpatient and outpatient	†	17	2 subscales: disease and treatment, and psychological	Three-point scale (I absolutely need to know, I would like to know, I do not want to know)
SST-IUPCN	English [42]	United States	2015	194 cancer patients	Inpatient and outpatient	†	11	5 dimensions: extent of disease, performance status, prognosis, comorbidities, and PC-specific problems	Total score ranges from 0 to 14
NEQ	Italian [43]	Italy	2000	423 patients with colon-rectum, genito-urinary, breast and other cancer	Inpatient	5 min	11	4 factors: informative needs about diagnosis and prognosis, informative needs about exams and treatments, communicative needs, and relational needs	Dichotomous (present vs absent)
	Italian [44]	Italy	2009	542 patients with gastrointestinal, hematological, respiratory and other tumors	Inpatient	10 min	22	5 factors: informative needs about diagnosis, prognosis and treatments, needs related to assistance/care, relational needs, needs for a psycho-emotional support, and material needs	Same response format as [52]
	Italian [45]	Italy	2016	783 patients with breast, lung, colon-rectum and other cancer	Inpatient and outpatient	†	23	5 factors: informative needs about diagnosis, prognosis and treatments, needs related to assistance/care, relational needs, needs for a psycho-emotional support, and material needs; 1 open question	Same response format as [52]

† No data available

SCNS-SF, Supportive care needs survey-short form, SCNS-ST9, Supportive care needs survey-screening tool, SCNAT-IP, Supportive cancer care needs assessment tool for Indigenous people, CANDI, Needs based biopsychosocial distress instrument for cancer patients, CARES, Cancer rehabilitation evaluation system, CARES-SF, Cancer rehabilitation evaluation system-short form, CaSUN, Cancer survivors’ unmet needs measure, SUNS, Survivors unmet needs survey, SUNS-SF, Survivors unmet needs survey-short form, SPARC, Sheffield profile for assessment and referral for care; NA-ACP, Needs assessment for advanced cancer patients, NA-ALCP, Needs assessment for advanced lung cancer patients, SPEED, Screen for palliative and end-of-life care needs in the emergency department, 3LNQ, Three-Levels-of-Needs questionnaire, CNQ-SF, Cancer needs questionnaire-short form, CNAT, Comprehensive needs assessment tool in cancer, PNPC, Problems and needs in palliative care questionnaire, ISQ, Information styles questionnaire, NEQ, Needs evaluation questionnaire, SST-IUPCN, Simple screening tool for identifying unmet palliative care needs

### Characteristics of the needs assessment tools

All of the 20 instruments were originally designed to measure comprehensive care needs among multiple cancer patients. Of those, 7 tools were originally developed in Australia (SCNS-SF, Supportive care needs survey screening tool-9 items: SCNS-ST9, Supportive cancer care needs assessment tool for Indigenous people: SCNAT-IP, CaSUN, Needs assessment for advanced cancer patients: NA-ACP, Needs assessment for advanced lung cancer patients: NA-ALCP and Cancer needs questionnaire-short form: CNQ-SF), [13,14,26,27,35,36,50] 5 in the United States (CANDI, Cancer rehabilitation evaluation system: CARES, Cancer rehabilitation evaluation system-short form: CARES-SF, Screen for palliative and end-of-life care needs in the emergency department: SPEED and Simple screening tool for identifying unmet palliative care needs: SST-IUPCN), [16,29,31,36,42] 2 in Canada (SUNS and Survivors unmet needs survey-short form: SUNS-SF), [15,33] and 1 in the United Kingdom (Sheffield profile for assessment and referral for care: SPAPC), [54] Denmark (Three-Levels-of-Needs questionnaire: 3LNQ), [37] South Korea (Comprehensive needs assessment tool in cancer: CNAT), [38] Netherland (Problems and needs in palliative care questionnaire: PNPC), [40] Greece (Information styles questionnaire: ISQ) [41] and Italy (Needs evaluation questionnaire: NEQ), [43] respectively. Moreover, 7 instruments were developed specifically for assessing care needs in advanced or palliative cancer patients (SPAPC, NA-ACP, NA-ALCP, SPEED, 3LNQ, PNPC and SST-IUPCN), [36–38,40,42,53,54] and 2 instruments performed as screening tools for the unmet care needs with fewer items and response burden (SCNS-ST9 and SST-IUPCN). [26,42] Among those, the SCNS-SF is the instrument with the most cross-cultural adaptations and the most tested measurement properties. It has been translated into a variety of language versions (e.g., French, German, Japanese, Traditional Chinese, Mandarin, Spanish and Dutch versions). [22–25,46–49] (Table 1)

The total number of items in each instrument ranged from 11 to 139, and all the evaluated instruments had a multi-dimensional structure. However, there were a great degree of variability in the content and construct in these recruited needs assessment tools. In sum, 8 health-status related and 5 health care-related domains were evaluated by 18 tools except for the 3LNQ without reporting specific needs assessment domains. [37] The majority of the instruments comprised physical, psychological, health care, information and communication domains. Only the SCNAT-IP assessed cultural issues, as it was designed specifically for indigenous people in Australia. [27] (Table 1)

Furthermore, as the most widely used tool, the SCNS-SF has been cross-culturally evaluated in several studies, its French, [46] German, [22] Japanese, [47] Traditional Chinese, [23] and Mandarin versions [24] showed the same 5 domains as the original English version, [13] whereas a Mandarin and Cantonese version and a Dutch version demonstrated that it was composed of 4 domains. [48,49] Moreover, although the Mandarin and Mexican-Spanish versions [24,25] had 5 subscales, a total of 33 items were included in the 2 translated versions which had a little difference with the original tool with 34 items. As for the NEQ, although 3 studies were performed in Italian cancer patients to test its psychometric properties, there was still a great deal of variability in its scale structure. [43–45] (Table 1)

In addition, a great degree of variability was discovered in response formats. The majority of the instruments adopted a five-point rating scale (SCNS-SF, [13,22–25,46–49] SCNS-ST9, [26] SCNAT-IP, [27] SUNS, [15,52] SUNS-SF, [33] NA-ACP, [35] and CNQ-SF [39]), or a four-point rating scale (SCNS-SF, [50] NA-ALCP, [53] and CNAT [38]), or dichotomous items [43–45] for response options to assess cancer care needs. Some tools adopted a combination of formats to accommodate different types of questions comprising scores for symptom distress and care needs (CANDI, [16] CARES, [29] CARES-SF, [31] SPARC, [54] 3LNQ, [37] and PNPC [40]). There were also fewer tools using a Likert-type scale to identify the degree to which a problem or symptom was experienced. [16,36,42] With regard to the completion time of these evaluated instruments, wide ranges of completion time were reported ranging from 5 min to 76 min, the respondents were required to spend over 30 min in filling out the CARES, [30] SPARC, [54] and NA-ACP, [35] which indicated a severe response burden (Table 1).

### Methodological quality of each study

Most of the studies assessed internal consistency, content validity, structural validity and hypothesis testing. Nevertheless, only one study tested responsiveness, and none of the studies assessed measurement error and criterion validity. Of the psychometric properties appraised, most of the studies were rated as excellent or good methodological quality in internal consistency (22/32, 68.8%) and structural validity (24/28, 85.7%), and fair methodological quality in reliability (12/13, 92.3%) and hypothesis testing (16/29, 55.2%). All of the studies were rated as excellent methodological quality in content validity. Moreover, one study assessing responsiveness was rated as fair methodological quality due to unclear hypotheses. The majority of studies that evaluated cross-cultural validity were rated as poor methodological quality because confirmatory factor analysis method (CFA) was not performed (12/13, 92.3%) (Table 2).

**Table 2:** Methodological quality of the studies on needs assessment tools in cancer patients.

Instrument	Language	Internal consistency	Reliability	Content validity	Structural validity	Hypothesis testing	Responsiveness	Cross-cultural validity
SCNS-SF	English [13]	good	†	excellent	good	good	†	†
	French [46]	excellent	good	excellent	excellent	good	†	good
	German [22]	good	†	excellent	good	good	†	poor
	Japanese [47]	good	†	excellent	good	good	†	poor
	Traditional Chinese [23]	good	†	excellent	good	good	†	†
	Mandarin [24]	good	†	excellent	good	good	†	poor
	Mandarin and Cantonese [48]	good	†	excellent	good	good	†	poor
	Spanish [25]	good	fair	excellent	good	fair	†	poor
	Dutch [49]	good	fair	excellent	good	good	†	poor
	English [50]	good	†	†	good	good	†	†
SCNS-ST9	English [26]	†	†	excellent	†	†	†	†
SCNAT-IP	English [27]	good	†	excellent	good	good	†	†
CANDI	English [16]	poor	fair	excellent	†	poor	†	†
	Turkish [28]	poor	fair	excellent	poor	fair	†	poor
CARES-SF	English [31]	good	fair	excellent	good	fair	fair	†
CARES	English [29]	poor	fair	excellent	†	fair	†	†
	Flemish [30]	poor	fair	excellent	poor	fair	†	poor
CaSUN	English [14]	good	fair	excellent	good	fair	†	†
	Dutch [32]	good	fair	excellent	good	fair	†	poor
	Chinese [51]	good	†	excellent	good	fair	†	poor
SUNS	English [15]	good	†	excellent	good	†	†	†
	English [52]	good	fair	excellent	good	fair	†	†
SUNS- SF	English [33]	good	†	excellent	good	fair	†	†
SPARC	English [54]	†	†	excellent	†	†	†	†
	Polish [34]	poor	†	excellent	poor	fair	†	poor
NA-ACP	English [35]	poor	fair	excellent	poor	†	†	†
NA-ALCP	English [53]	poor	†	excellent	†	good	†	†
SPEED	English [36]	poor	†	excellent	†	†	†	†
3LNQ	Danish [37]	†	†	excellent	†	†	†	†
CNAT	Korean [38]	good	†	excellent	good	fair	†	†
CNQ-SF	English [39]	good	†	†	good	good	†	†
PNPC	Dutch [40]	poor	†	excellent	†	fair	†	†
ISQ	Greek [41]	good	†	excellent	good	fair	†	poor
SST-IUPCN	English [42]	poor	†	excellent	†	fair	†	†
NEQ	Italian [43]	good	fair	excellent	good	†	†	†
	Italian [44]	†	†	†	good	†	†	†
	Italian [45]	†	†	†	good	fair	†	†

† No data available

### The quality of psychometric properties

The reliability, responsiveness and validity assessment of the included instruments were presented in S3 and S4 Tables, respectively. Regarding the quality of measurement properties, internal consistency was evaluated in 32 studies with 9 studies showing negative rating [16,23,31,32,34,40,43,51,53] and 4 studies indeterminate rating. [28,29,36,42] Less than half of the studies assessed test-retest reliability (13/37, 35.1%), and only 4 studies showed positive scoring. [16,25,28,49] No data was available on measurement error. As for the validity assessment, content validity was the most tested psychometric property which was evaluated in 33 studies, and showed positive rating in all of the evaluated studies. Structural validity was adequately evaluated in 28 studies with 5 studies showing positive rating. [23,43–45,46] More than half of the studies tested hypothesis testing (29/37, 78.4%), and 16 studies were rated intermediate as no hypotheses were developed before data collection. [14,16,25,28–34,38,40–42,45,51] Cross-cultural validity results were obtained in 13 studies with 7 studies indicating negative rating. [28,30,32–34,48,49] No information was found on criterion validity. In addition, only one study tested responsiveness and showed intermediate scoring (Table 3). [31]

**Table 3:** Quality of each psychometric property of needs assessment tools in cancer patients.

Instrument	Language	Internal consistency	Test-retest reliability	Content validity	Structural validity	Hypothesis testing	Responsiveness	Cross-cultural validity
SCNS-SF	English [13]	+	0	+	?	+	0	0
	French [46]	+	?	+	+	+	0	+
	German [22]	+	0	+	?	+	0	+
	Japanese [47]	+	0	+	?	+	0	+
	Traditional Chinese [23]	-	0	+	+	+	0	0
	Mandarin [24]	+	0	+	?	+	0	+
	Mandarin and Cantonese [48]	+	0	+	?	+	0	-
	Spanish [25]	+	+	+	?	?	0	+
	Dutch [49]	+	+	+	?	+	0	-
	English [50]	+	0	0	?	+	0	0
SCNS-ST9	English [26]	0	0	+	0	0	0	0
SCNAT-IP	English [27]	+	0	+	?	+	0	0
CANDI	English [16]	-	+	+	0	?	0	0
	Turkish [28]	?	+	+	?	?	0	-
CARES-SF	English [31]	-	?	+	?	?	?	0
CARES	English [29]	?	?	+	0	?	0	0
	Flemish [30]	+	?	+	?	?	0	-
CaSUN	English [14]	+	?	+	?	?	0	0
	Dutch [32]	-	-	+	?	?	0	-
	Chinese [51]	-	0	+	?	?	0	-
SUNS	English [15]	+	0	+	?	0	0	0
	English [52]	+	-	+	?	+	0	0
SUNS- SF	English [33]	+	0	+	?	?	0	0
SPARC	English [54]	0	0	+	0	0	0	0
	Polish [34]	-	0	+	?	?	0	-
NA-ACP	English [35]	+	-	+	?	0	0	0
NA-ALCP	English [53]	-	0	+	0	+	0	0
SPEED	English [36]	?	0	+	0	0	0	0
3LNQ	Danish [37]	0	0	+	0	0	0	0
CNAT	Korean [38]	+	0	+	?	?	0	0
CNQ-SF	English [39]	+	0	0	?	+	0	0
PNPC	Dutch [40]	-	0	+	0	?	0	0
ISQ	Greek [41]	+	0	+	?	?	0	+
SST-IUPCN	English [42]	?	0	+	0	?	0	0
NEQ	Italian [43]	-	-	+	+	0	0	0
	Italian [44]	0	0	0	+	0	0	0
	Italian [45]	0	0	0	+	?	0	0

+, positive rating, ?, indeterminate rating, -, negative rating, 0, no data available

### Evidence synthesis

All of the recruited tools reported strong evidence for content validity. About half of the instruments had strong or moderate evidence for internal consistency and structural validity. Only one instrument had moderate evidence for cross-cultural validity. In sum, SCNS-SF had strong evidence for 6 measurement properties (internal consistency, reliability, content validity, structural validity, hypothesis testing and cross-cultural validity), and moderate evidence for cross-cultural validity. [13,22–25,46–50] SCNAT-IP had strong evidence for content validity, and moderate evidence for internal consistency, structural validity and hypothesis testing. [27] CaSUN also reported strong or moderate evidence for 5 measurement properties (internal consistency, reliability, content validity, structural validity and hypothesis testing) (Table 4). [14,32,33]

**Table 4:** Evidence synthesis of needs assessment tools in cancer patients.

Instrument	Internal consistency	Reliability	Content validity	Structural validity	Hypothesis testing	Responsiveness	Cross-cultural validity
SCNS-SF [13,22–25,46–50]	†††	††	†††	†††	†††		††
SCNS-ST9 [26]			†††				
SCNAT-IP [27]	††		†††	††	††		
CANDI [16,28]	?	††	†††	?	†		?
CARES-SF [31]	††	†	†††	††	†	†	
CARES [29,30]	?	††	†††	?	††		?
CaSUN [14,32,51]	†††	††	†††	†††	††		?
SUNS [15,52]	†††	†	†††	†††	†		
SUNS- SF [33]	††		†††	††	†		
SPARC [34,54]	?		†††	?	†		?
NA-ACP [35]	?	†	†††	?			
NA-ALCP [53]	?		†††		††		
SPEED [36]	?		†††				
3LNQ [37]			†††				
CNAT [38]	††		†††	††	†		
CNQ-SF [39]	††			††	††		
PNPC [40]	?		†††		†		
ISQ [41]	††		†††	††	†		?
SST-IUPCN [42]	?		†††		†		
NEQ [43–45]	††	†	†††	†††	†		

†††, strong evidence

††, moderate evidence

†, limited evidence

?, unknown

## Discussion

This study has conducted a systematic assessment on the psychometric properties of needs assessment tools for cancer patients according to the agreed quality criteria for measurement properties of health status questionnaires. [17] Despite a previous literature review has focused on needs assessment tools (1984–2004) for cancer patients, [7] a scoring system was not used for the assessment of measurement properties. In addition, several novel needs assessment tools for cancer patients have been developed in recent years, which make it essential to carry out an updated systematic review on the psychometric properties of cancer-specific needs assessment tools and make recommendations on the most appropriate instruments in clinical practice.

It should be noted that, the instruments identified in the review are not site specific, and are general measures for the patients with the most common solid tumors. Thus, certain site-specific care needs such as body image management in breast cancer, and difficulties in swallowing and chewing in head and neck cancer are not highlighted. For needs assessment of these specific tumors, it may be beneficial to developing site-specific modules (e.g., head and neck cancer-specific version) [55] as a supplement, which is similar to the evaluation of HRQOL. Moreover, it was found that, despite the physical, psychological, health care, information and communication needs are the most common domains in these needs assessment tools, a great deal of variability still exists in the content and structure. This may be ascribed to the subjective nature of instrument development in the interpretation of qualitative data from cancer patients and experts, and the adoption of different factor analysis method, as well as a lack of a conceptual framework. [11] In addition, our concern was to identify the tools for needs evaluation in cancer patients. However, certain instruments have focused on identifying the extent to the actual problems and symptoms. Therefore, a lack of discrimination between the appraisal of perceived symptoms and the needs for receiving care may cause ambiguity about whether or not the individuals want assistance.

This review has identified 37 studies evaluating the psychometric properties of 20 instruments. However, none of the studies assessed all of the measurement properties Terwee et al. (2007) has recommended. [17] Furthermore, despite the agreed quality criteria for measurement properties of health status questionnaires was developed in 2007, [17] 29 studies performed after the publication of the quality criteria did not follow this guideline, [13,15,16,22–28,46–50,30,32–34,36–38,41,42,44,45,51–53] which suggested that higher methodological quality studies on instrument development for cancer-specific care needs assessment should be highlighted in future studies.

The review showed that, none of the studies tested measurement error. Measurement error refers to the systematic and random error of a patient’s score that is not attributed to true changes in the construct to be measured. [21] Based on the COSMIN checklist, measurement error is regarded as one of the important reliability measurement properties, and is rated as positive if the minimal important change (MIC) is larger than the smallest detectable change (SDC), or the MIC is outside the limits of agreement (LOA). [21] As a result, in future studies, it is beneficial to incorporating measurement error as one of the measurement properties.

Moreover, responsiveness is defined as the ability of a health-related patient-reported outcome (HR-PRO) instrument to detect clinically important change over time in the construct to be measured. [20] Therefore, it is helpful for health care professionals to adopt an instrument with acceptable psychometric properties during and after treatment that are responsive to clinical changes. Nonetheless, minimal attention (one study) was given to the psychometric property. [31] Instruments that can detect clinical change over time allow for comparisons across different time points. [56] In addition, it was found that criterion validity was not tested in all of the studies, which may be ascribed the fact that there is still a lack of an adequate gold standard for comparison in care needs among cancer patients.

The review also showed that, despite the majority of the studies appraised internal consistency, some were rated negative, as Cronbach’s alpha for at least one or more subscales was lower than 0.70. [17,21] The alpha value is determined by the number of items, item interrelatedness and dimensionality. [57] A low alpha can be ascribed to a low number of items, poor item interrelatedness or heterogeneous constructs. Therefore, a low alpha in subscales of the recruited tools suggests that some items should be revised or discarded, and the easiest method is to compute the item-total score correlation and delete items with low correlations. [58] Moreover, four studies showed indeterminate rating in internal consistency because structural validity was insufficient or only the overall Cronbach’s alpha for the whole scale was computed. [28,29,36,42] The results suggest that, for the analysis of internal consistency, factor analysis is needed to check the dimensionality of the scale and Cronbach’s alpha of each subscale is required to calculated separately in future studies.

Test-retest reliability is a critical measurement property for the assessment of instrument stability with different time interval. In the review, only about one-third of the studies tested test-retest reliability, and the majority of these studies reported negative or intermediate rating, as ICC or weighted Kappa for at least one or more subscales was < 0.70 or was not reported. Furthermore, although positive rating of test-retest reliability was given to 4 studies, [16,25,28,49] there was a great degree of variability in time interval ranging from 3 to 28 days, which may affect the methodological quality of the measurement property.

Moreover, regarding the validity appraisal, most of the studies evaluated structural validity. Twenty-three studies were classified as indeterminate because CFA method was not used, or related fit indexes such as Comparative Fit Index (CFI) and Root Mean Square Error of Approximation (RMSEA) were not calculated. [21] As for the quality criteria for structural validity, Terwee (2011) proposes that factors which explain at least 50% of the variance is rated as positive, no matter what types of methods are used (exploratory factor analysis-EFA or CFA). [59] However, in 2017, Mokkink et al. (2017) highlights that structural validity is rated as positive, when CFA is conducted with CFI or TLI or comparable measure > 0.95 or RMSEA <0.06 or SRMR < 0.08. [21] Furthermore, although approximately two-third of the studies tested hypothesis testing, about half of the studies were rated indeterminate as no related hypotheses were defined in advance. The results indicate that, in order to improve the methodological quality of hypothesis testing, it is beneficial to developing multiple hypotheses regarding correlations or mean differences before data collection in future studies.

In addition, cross-cultural validity was tested in 13 studies with 7 studies showing negative rating due to differences in factor structure. [28,30,32,34,48,49,51] The findings suggested that, cross-cultural adaptability and feasibility of the recruited tools were insufficient. Meanwhile, although these cross-cultural studies adopted a rigorous research design and translation procedure, the methodological quality was still poor in many translated versions, which may be ascribed to the methodological deficiency with no CFA performed or insufficient sample size, which was recommended in the COSMIN checklist. [19] Therefore, these instruments may benefit from further cross-cultural validation using more appropriate factor analysis method (CFA) and including more samples.

The strengths of the systematic review are the adoption of the COSMIN checklist and criteria for evidence synthesis which ensured that the appraisal of the recruited tools was robust and rigorous. However, several limitations have been noted. First, only studies with English language were eligible in the review, which may cause selection bias. More articles published in other languages are recommended to be recruited in future reviews. Second, although we have contacted the authors of the original studies for missing data, most of whom did not respond, which might lead to exclusion of these incomplete papers. Third, the search was constrained to the most used electronic databases, which might inevitably result in missing publications and publication bias. Finally, the recruited studies did not evaluate a number of psychometric properties sufficiently such as measurement error and responsiveness, which could make it difficult and sometimes impossible to test these properties.

## Conclusions

In summary, the systematic review has focused on needs assessment tools that have not been fully investigated among cancer patients. SCNS-SF is the most widely used instrument for needs assessment. It had strong evidence for internal consistency, reliability, content validity, structural validity and hypothesis testing, and moderate evidence for cross-cultural validity. CaSUN reported strong or moderate evidence for internal consistency, reliability, content and structural validity, and hypothesis testing. Moreover, SCNAT-IP had strong evidence for content validity, and moderate evidence for internal consistency, structural validity and hypothesis testing. Nonetheless, none of the studies assessed measurement error and only one study tested responsiveness. Further improvement of already existing and promising measurements is recommended. It is essential for oncology health care professionals to select the most appropriate instruments for needs evaluation among cancer patients. Their appropriate selection and use of these instruments will be beneficial to early identification and effective cancer care management.

## Supporting information

S1 Appendix Search strategy. (DOCX)

S2 Appendix Full names of the instruments and their abbreviations. (DOCX)

S3 Appendix PRISMA checklist. (DOC)

S1 Table Quality criteria for measurement properties. (DOCX)

S2 Table Levels of evidence for the quality of the measurement property. (DOCX)

S3 Table Reliability assessment of needs assessment tools in cancer patients. (DOCX)

S4 Table Validity assessment of needs assessment tools in cancer patients. (DOCX)
