# Supplementary material for: Evaluation of psychometric properties of needs assessment tools in cancer patients: A systematic literature review
Source: PLoS One. 2019 Jan 8;14(1):e0210242. doi: 10.1371/journal.pone.0210242 (PMC6324833; doi:10.1371/journal.pone.0210242)
Supplement: S1 Appendix — (DOCX) [file pone.0210242.s001.docx]

## **S1 Appendix: Search strategy**

## **Pubmed (From 1966 to 08/31/2018)**

#1. (neoplasm*[tiab] OR cancer*[tiab] OR carcinoma*[tiab] OR tumor*[tiab] OR oncology*[tiab] OR malignan*[tiab] OR lymphoma[tiab] OR melanoma[tiab] OR leukemia[tiab] OR sarcoma[tiab]) OR (neoplasms) [MeSH]

#2. (evaluation*[tiab] OR assessment*[tiab] OR psychometric*[tiab] OR measure*[tiab] OR propert*[tiab] OR develop*[tiab] OR reliab*[tiab] OR valid*[tiab] OR responsive*[tiab] OR method*[tiab] OR tool*[tiab] OR instrument*[tiab] OR scale*[tiab] OR survey*[tiab] OR questionnaire*[tiab] OR version*[tiab]) AND need*[tiab]

#3. (needs assessment) [MeSH]

#4. #2 OR #3

#5. #1 AND #4

**CINAHL (From 1960** **to 08/31/2018)**

S1. TI “neoplasm” OR AB “neoplasm” OR TI “cancer” OR AB “cancer” OR TI “carcinoma” OR AB “carcinoma” OR TI “tumor” OR AB “tumor” OR TI “oncology” OR AB “oncology” OR TI “malignancy” OR AB “malignancy” OR TI “lymphoma” OR AB “lymphoma” OR TI “melanoma” OR AB “melanoma” OR TI “leukemia” OR AB “leukemia” OR TI “sarcoma” OR AB “sarcoma” OR MH “neoplasms”

S2. TI “evaluation” OR AB “evaluation” OR TI “assessment” OR AB “assessment” OR TI “psychometric*” OR AB “psychometric*” OR TI “measure*” OR AB “measure*” OR TI “propert*” OR AB “propert*” OR TI “develop*” OR AB “develop*” OR TI “reliab*” OR AB “reliab*” OR TI “valid*” OR AB “valid*” OR TI “responsive*” OR AB “responsive*” OR TI “method*” OR AB “method*” OR TI “tool*” OR AB “tool*” OR TI “instrument*” OR AB “instrument*” OR TI “scale*” OR AB “scale*” OR TI “survey*” OR AB “survey*” OR TI “questionnaire*” OR AB “questionnaire*” OR TI “version*” OR AB “version*”

S3. MH “needs assessment” OR TI “need*” OR AB “need*”

S4. S1 AND S2 AND S3

**Embase (From 1980** **to 08/31/2018)**

1. neoplasm. ti,ab. OR cancer. ti,ab. OR carcinoma. ti,ab. OR tumor. ti,ab. OR oncology. ti,ab. OR malignancy. ti,ab. OR lymphoma. ti,ab. OR melanoma. ti,ab. OR leukemia. ti,ab. OR sarcoma. ti,ab.

2. evaluation. ti,ab. OR assessment. ti,ab. OR psychometric. ti,ab. OR measure. ti,ab. OR measurement. ti,ab. OR property. ti,ab. OR properties. ti,ab. OR development. ti,ab. OR develop. ti,ab. OR reliability. ti,ab. OR validity. ti,ab. OR responsiveness. ti,ab. OR method. ti,ab. OR methodology. ti,ab. OR tool. ti,ab. OR instrument. ti,ab. OR scale. ti,ab. OR survey. ti,ab. OR questionnaire. ti,ab. OR version. ti,ab.

3. need*. ti,ab.

4. 1 AND 2 AND 3

**PsychINFO (From 1967 to 08/31/2018)**

1. TI “neoplasm” OR AB “neoplasm” OR TI “cancer” OR AB “cancer” OR TI “carcinoma” OR AB “carcinoma” OR TI “tumor” OR AB “tumor” OR TI “oncology” OR AB “oncology” OR TI “malignancy” OR AB “malignancy” OR TI “lymphoma” OR AB “lymphoma” OR TI “melanoma” OR AB “melanoma” OR TI “leukemia” OR AB “leukemia” OR TI “sarcoma” OR AB “sarcoma”

2. TI “evaluation” OR AB “evaluation” OR TI “assessment” OR AB “assessment” OR TI “psychometric*” OR AB “psychometric*” OR TI “measure*” OR AB “measure*” OR TI “propert*” OR AB “propert*” OR TI “develop*” OR AB “develop*” OR TI “reliab*” OR AB “reliab*” OR TI “valid*” OR AB “valid*” OR TI “responsive*” OR AB “responsive*” OR TI “method*” OR AB “method*” OR TI “tool*” OR AB “tool*” OR TI “instrument*” OR AB “instrument*” OR TI “scale*” OR AB “scale*” OR TI “survey*” OR AB “survey*” OR TI “questionnaire*” OR AB “questionnaire*” OR TI “version*” OR AB “version*”

3. TI “need*” OR AB “need*”

4. 1 AND 2 AND 3

**Google scholar (From inception to 08/31/2018)**

abstract: ("neoplasm" OR "cancer" OR "carcinoma" OR "tumor") AND ("need" or "needs") AND ("reliability" OR "validity" OR "evaluation" OR "assessment" OR "psychometrics" OR "measurement" OR "properties" OR "questionnaire" OR "scale" OR "instrument")
