# Supplementary material for: Evaluation of psychometric properties of needs assessment tools in cancer patients: A systematic literature review
Source: PLoS One. 2019 Jan 8;14(1):e0210242. doi: 10.1371/journal.pone.0210242 (PMC6324833; doi:10.1371/journal.pone.0210242)
Supplement: S2 Appendix — (DOCX) [file pone.0210242.s002.docx]

## **S2 Appendix: Full names of the instruments and their abbreviations**

| **Full names of the instruments** | **Abbreviations** |
| --- | --- |
| Supportive care needs survey-short form | SCNS-SF |
| Supportive care needs survey-screening tool-9 items | SCNS-ST9 |
| Supportive cancer care needs assessment tool for Indigenous people | SCNAT-IP |
| Needs based biopsychosocial distress instrument for cancer patients | CANDI |
| Cancer rehabilitation evaluation system | CARES |
| Cancer rehabilitation evaluation system-short form | CARES-SF |
| Cancer survivors’ unmet needs measure | CaSUN |
| Survivors unmet needs survey | SUNS |
| Survivors unmet needs survey-short form | SUNS-SF |
| Sheffield profile for assessment and referral for care | SPARC |
| Needs assessment for advanced cancer patients | NA-ACP |
| Needs assessment for advanced lung cancer patients | NA-ALCP |
| Screen for palliative and end-of-life care needs in the emergency department | SPEED |
| Three-Levels-of-Needs questionnaire | 3LNQ |
| Cancer needs questionnaire-short form | CNQ-SF |
| Comprehensive needs assessment tool in cancer | CNAT |
| Problems and needs in palliative care questionnaire | PNPC |
| Information styles questionnaire | ISQ |
| Needs evaluation questionnaire | NEQ |
| Simple screening tool for identifying unmet palliative care needs | SST-IUPCN |
