# Supplementary material for: Evaluation of psychometric properties of needs assessment tools in cancer patients: A systematic literature review
Source: PLoS One. 2019 Jan 8;14(1):e0210242. doi: 10.1371/journal.pone.0210242 (PMC6324833; doi:10.1371/journal.pone.0210242)
Supplement: S1 Table — (DOCX) [file pone.0210242.s004.docx]

**S1 Table. Quality criteria for measurement properties**

| **Measurement property** | **Rating ^1^** | **Quality criteria** |
| --- | --- | --- |
| **Reliability** |  |  |
| Internal consistency | + | At least low evidence^2^ for sufficient structural validity^3^ AND Cronbach's alpha(s) ≥ 0.70 for each unidimensional scale or subscale^4^ |
|  | ? | Criteria for “At least low evidence^2^ for sufficient structural validity^3^” not met |
|  | - | At least low evidence^2^ for sufficient structural validity^3^ AND Cronbach’s alpha(s) < 0.70 for each unidimensional scale or subscale^4^ |
| Test-retest reliability | + | ICC or weighted Kappa ≥ 0.70 |
|  | ? | ICC or weighted Kappa not reported |
|  | - | ICC or weighted Kappa < 0.70 |
| Measurement error | + | SDC or LoA < MIC^3^ |
|  | ? | MIC not defined |
|  | - | SDC or LoA > MIC^3^ |
| **Validity** |  |  |
| Content validity | + | A clear description is provided of the measurement aim, the target population,  the concepts that are being measured, and the item selection AND target population  and (investigators OR experts) were involved in item selection |
|  | ? | A clear description of above-mentioned aspects is lacking OR only target population involved OR doubtful design or method |
|  | - | No target population involvement |
| Structural validity | + | CTT:  CFA: CFI or TLI or comparable measure > 0.95 OR RMSEA <0.06 OR SRMR < 0.08^5^ |
|  | ? | CTT: Not all information for ‘+’ reported |
|  | - | Criteria for ‘+’ not met |
| Hypothesis testing | + | The result is in accordance with the hypothesis^6^ |
|  | ? | No hypothesis defined (by the review team) |
|  | - | The result is not in accordance with the hypothesis^6^ |
| Cross-cultural validity | + | No important differences found between group factors (such as age, gender, language) in multiple group factor analysis OR no important DIF for group factors (McFadden's R^2^ < 0.02) |
|  | ? | No multiple group factor analysis OR DIF analysis performed |
|  | - | Important differences between group factors OR DIF was found |
| Criterion validity | + | Correlation with gold standard ≥ 0.70 OR AUC ≥ 0.70 |
|  | ? | Not all information for ‘+’ reported |
|  | - | Correlation with gold standard < 0.70 OR AUC < 0.70 |
| **Responsiveness** |  |  |
| Responsiveness | + | The result is in accordance with the hypothesis^6^ OR AUC ≥ 0.70 |
|  | ? | No hypothesis defined (by the review team) |
|  | - | The result is not in accordance with the hypothesis^6^ OR AUC < 0.70 |

The criteria are from: https://cosmin.nl/wp-content/uploads/COSMIN_manual_syst-review-PROMs_V1.0.pdf

AUC, area under the curve, CFA, confirmatory factor analysis, CFI, comparative fit index, CTT, classical test theory, DIF, differential item functioning, ICC, intraclass correlation coefficient, LoA, limits of agreement, MIC, minimal important change, RMSEA, Root Mean Square Error of Approximation, SDC, smallest detectable change, SRMR, Standardized Root Mean Residuals, TLI, Tucker-Lewis index

1 “+” = sufficient, ” –“ = insufficient, “?” = indeterminate

2 As defined by grading the evidence according to the GRADE approach

3 This evidence may come from different studies

4 The criteria ‘Cronbach alpha < 0.95’ was deleted, as this is relevant in the development phase of a PROM and not when evaluating an existing PROM

5 To rate the quality of the summary score, the factor structures should be equal across studies

6 The results of all studies should be taken together and it should then be decided if 75% of the results are in accordance with the hypotheses
