# Supplementary material for: Evaluation of psychometric properties of needs assessment tools in cancer patients: A systematic literature review
Source: PLoS One. 2019 Jan 8;14(1):e0210242. doi: 10.1371/journal.pone.0210242 (PMC6324833; doi:10.1371/journal.pone.0210242)
Supplement: S2 Table — (DOCX) [file pone.0210242.s005.docx]

| **S2 Table. Levels of evidence for the quality of the measurement property** | | |
| --- | --- | --- |
| **Level** | **Rating*** | **Criteria** |
| Strong | +++ or --- | Consistent findings in multiple studies of good methodological quality OR in one study of excellent methodological quality |
| Moderate | ++ or -- | Consistent findings in multiple studies of fair methodological quality OR in one study of good methodological quality |
| Limited | + or - | One study of fair methodological quality |
| Conflicting | +/- | Conflicting findings |
| Unknown | ? | Only studies of poor methodological quality |
|  |  |  |

+, positive rating, ?, indeterminate rating, -, negative rating

| Appendix 4. Description of Eligible Study Populations | | | | | | |  |  |  |  |  |
| --- | --- | --- | --- | --- | --- | --- | --- | --- | --- | --- | --- |
| Reference | Evaluated instrument(s) | Year of Publication | Country | Language | Study Population | Total Sample Size (thyroid cancer) | Cancer staging | Thyroidectomy | Mean TSH in mU/L (SD) | Mean age (SD) | Male |
| [[32](#_ENREF_32)] | ThyDQoL and ThyTSQ | 2004 | UK | English | Hypothyroidism | 38 (0) | Nil | 23.7% | NR | 51.9 (14.8) | 21.1% |
| [[33](#_ENREF_33)] | ThyDQoL and ThySRQ | 2008 | UK | English | Hypothyroidism | 110 (9) | NR | NR | 3.9 (5.0) | 55.1 (14.3) | 19.1% |
| [[34](#_ENREF_34)] | ThyDQoL, ThyTSQ and ThySRQ | 2013 | Germany | German | Hypothyroidism | 101 (0) | Nil | 14.9% | 2.20 (3.95) | 43.5 (16.3) | 7.8% |
| [[35](#_ENREF_35)] | ThyTSQ | 2006 | UK | English | Hypothyroidism | 103 (9) | NR | NR | 3.8 (5.2) | 55.2 (14.4) | 17.5% |
| [[38](#_ENREF_38)] | HCQ | 2000 | Netherlands | Dutch | Hyperthyroidism | 303 (0) | Nil | NR | NR | 44.0 (11.2) | 12.8% |
| [[41](#_ENREF_41)] | WSCI-T | 2011 | Canada | English | Pre-thyroidectomy patients | 148 (27) | NR | 100% | NR | 49.9 (13.6) | 16.2% |
| [[28](#_ENREF_28)] | GO-QOL | 1998 | Netherlands | Dutch | Graves' ophthalmopathy | 70 (0) | Nil | NR | NR | 53.3 (13.1) | 28.6% |
| [[29](#_ENREF_29)] | GO-QOL | 1999 | Netherlands | Dutch | Graves' ophthalmopathy | 93 (0) | Nil | 15% | NR | 49.1 (13.7) | 24.7% |
| [[30](#_ENREF_30)] | GO-QOL | 2001 | Netherlands | Dutch | Graves' ophthalmopathy | 164 (0) | Nil | NR | NR | 50 (12) | 18.9% |
| [[31](#_ENREF_31)] | GO-QOL | 2012 | Korea | Korean | Graves' ophthalmopathy | 98 (0) | Nil | 5% | NR | 40.7 (NR) | 19.4% |
| [[39](#_ENREF_39)] | GO-QLS and NEI VFQ-25 | 2005 | USA | English | Graves' ophthalmopathy | 256 (0) | Nil | NR | NR | 57.14 (21.6) | 16.8% |
| [[40](#_ENREF_40)] | NEI VFQ-25 | 2006 | USA | English | Graves' ophthalmopathy | 30 (0) | Nil | NR | NR | 54† (20–73) | 26.7% |
| [[36](#_ENREF_36)] | TED-QOL, GO-QOL and GO-QLS | 2011 | Canada | English | Graves' ophthalmopathy | 100 (0) | Nil | NR | NR | <25: 8% 25-50: 42% >50: 50% | 20.0% |
| [[37](#_ENREF_37)] | TED-QOL | 2014 | Korea | Korean | Graves' ophthalmopathy | 90 (0) | Nil | 11.1% | 8.5 (10.4) | 42.9 (13.6) | 27.8% |
| [[21](#_ENREF_21)] | EORTC QLQ-C30 (Version 3) and QLQ-H&N35 | 2013 | Mexico | Spanish | Head and neck cancer including tumor location of thyroid carcinoma invasive to aerodigestive tract | 193 (32) | T1: 7.8% T2: 22.8% T3: 26.9% T4: 39.4% Unknown: 3.1% | NR | NR | 56.9 (NR) | 53.9% |
| [[42](#_ENREF_42)] | MDASI-THY | 2008 | USA | English | Thyroid cancer patients undergoing hormonal therapy | 60 (60) | Stage I: 18.3% Stage II: 5.0% Stage III: 11.7% Stage IV: 20.0% Unknown: 45% | NR | NR | 51.0 (13.2) | 45.0% |
| [[22](#_ENREF_22)] | ThyPRO | 2008 | Denmark | Danish | Thyroid disease patients including thyroid swelling and dysfunction of the gland | 31 (NR) | NR | NR | NR | 53† (26–76) | 6.5% |
| [[23](#_ENREF_23)] | ThyPRO | 2009 | Denmark | Danish | Benign thyroid disease | 907 (NR) | NR | 14.6% | NR | 51 (15) | 13.2% |
| [[24](#_ENREF_24)] | ThyPRO | 2010 | Denmark | Danish | Benign thyroid disease | 907 (NR) | NR | 14.6% | NR | 51 (15) | 13.2% |
| [[26](#_ENREF_26)] | ThyPRO | 2014 | Denmark | Danish | Benign thyroid disease | 907 (NR) | NR | 14.6% | NR | 51 (15) | 13.2% |
| [[25](#_ENREF_25)] | ThyPRO | 2014 | Denmark | Danish | Benign thyroid disease | 435 (NR) | NR | 23.2% | 0.42 (NR) | 54 (NR) | 17.0% |
| [[27](#_ENREF_27)] | ThyPRO | 2014 | Denmark | Danish | Benign thyroid disease | 838 (NR) | NR | NR | NR | 18-29: 7% 30-39: 18% 40-49: 21% 50-59: 25% 60-69: 19% >70: 10% >50: 50% | 13.1% |
| [[43](#_ENREF_43)] | THYCA-QoL | 2013 | Netherlands | Dutch | Thyroid cancer survivor | 306 (306) | Stage I: 56.2% Stage II: 19.3% Stage III: 15.7% Stage IV: 6.5% Unknown: 2.3% | 99.3% | NR | 56.4 (14.5) | 24.8% |
| Note: NR, Not Reported | |  |  |  |  |  |  |  |  |  |  |
| † Median is shown only | |  |  |  |  |  |  |  |  |  |  |
