# Supplementary material for: Evaluation of psychometric properties of needs assessment tools in cancer patients: A systematic literature review
Source: PLoS One. 2019 Jan 8;14(1):e0210242. doi: 10.1371/journal.pone.0210242 (PMC6324833; doi:10.1371/journal.pone.0210242)
Supplement: S3 Table — (DOCX) [file pone.0210242.s006.docx]

**S3 Table. Reliability assessment of needs assessment tools in cancer patients**

| Instrument | Language | Reliability | | | | Responsiveness | |
| --- | --- | --- | --- | --- | --- | --- | --- |
|  |  | Internal Consistency | Test-retest Reliability | | | Method | Results |
|  |  | Cronbach’s alpha | Time interval | Method | Result |  |  |
| SCNS-SF | English [13] | α, 0.87-0.96 | † | † | † | † | † |
|  | French [46] | α, 0.80-0.93 | 2 weeks | Pearson correlation | Physical and daily living needs, r=0.62, other scales, r>0.70 | † | † |
|  | German [22] | α, 0.82-0.95 | † | † | † | † | † |
|  | Japanese [47] | α, 0.86-0.96 | † | † | † | † | † |
|  | Traditional Chinese [23] | α, 0.53-0.89 (Hong Kong)  α, 0.76-0.97 (Taiwan) | † | † | † | † | † |
|  | Mandarin [24] | α, 0.85-0.95 | † | † | † | † | † |
|  | Mandarin and Cantonese [48] | α, 0.75-0.92 | † | † | † | † | † |
|  | Spanish [25] | α, 0.78-0.90 | 5 days | ICC | Overall, ICC=0.90 | † | † |
|  | Dutch [49] | α, 0.79-0.96 | 1 month | ICC | ICC, 0.74-0.83 | † | † |
|  | English [50] | α, 0.82-0.96 | † | † | † | † | † |
| SCNS-ST9 | English [26] | † | † | † | † | † | † |
| SCNAT-IP | English [27] | α, 0.82-0.95 | † | † | † | † | † |
| CANDI | English [16] | α, 0.60-0.92 | 3-7 days | ICC | Overall, ICC>0.99 | † | † |
|  | Turkish [28] | Overall, α=0.94 | 3-28 days | ICC | Overall, ICC=0.87 | † | † |
| CARES-SF | English [31] | Sample 1, α, 0.67-0.92  Sample 2, α, 0.67-0.85  Sample 3, α, 0.61-0.84 | 10 days | Pearson correlation | r, 0.69-0.92 | 3 time points (1mo, 7mo and 13mo) after primary surgery | Global CARES score improved from 1mo to 13mo after surgery  Physical and psychosocial summary scales showed change between all points |
| CARES | English [29] | Sample 1, α, 0.88-0.94  Sample 2, α, 0.82-0.94 | 10 days | Pearson correlation | r, 0.82-0.91 | † | † |
|  | Flemish [30] | α, 0.88-0.96 | 12.62 (9.3) days | Spearman correlation | r, 0.70-0.92 | † | † |
| CaSUN | English [14] | Overall, α=0.96, each factor, α, 0.78-0.93 | 3 weeks | Pearson correlation | Overall, r=0.18 | † | † |
| CaSUN-NL | Dutch [32] | α, 0.38-0.94 | 6 weeks | Kappa | 0.22-0.37 | † | † |
| CaSUN-C | Chinese [51] | Overall, α=0.87, each factor, α, 0.61-0.82 | † | † | † | † | † |
| SUNS | English [15] | α, 0.93-0.99 | † | † | † | † | † |
|  | English [52] | α, 0.92-0.99 | 7-14 days | ICC | ICC, 0.61-0.77 | † | † |
| SUNS-SF | English [33] | α, 0.85-0.95 | † | † | † | † | † |
| SPARC | English [54] | † | † | † | † | † | † |
|  | Polish [34] | α, 0.51-0.88 | † | † | † | † | † |
| NA-ACP | English [35] | α, 0.79-0.98 | 1 week | ICC | ICC, 0.67-0.93 | † | † |
| NA-ALCP | English [53] | α, 0.57-0.95 | † | † | † | † | † |
| SPEED | English [36] | α, 0.72-0.99 | † | † | † | † | † |
| 3LNQ | Danish [37] | † | † | † | † | † | † |
| CNAT | Korean [38] | α, 0.80-0.97 | † | † | † | † | † |
| CNQ-SF | English [39] | α, 0.77-0.94 | † | † | † | † | † |
| PNPC | Dutch [40] | α in problem aspect, 0.67-0.89  α in need for care aspect, 0.73-0.92 | † | † | † | † | † |
| ISQ | Greek [41] | α, 0.89-0.92 | † | † | † | † | † |
| SST-IUPCN | English [42] | Overall, α=0.81 | † | † | † | † | † |
| NEQ | Italian [43] | α, 0.69-0.81 | 1 week | Cohen's Kappa | Cohen's Kappa, 0.46-0.93 | † | † |
|  | Italian [44] | † | † | † | † | † | † |
|  | Italian [45] | † | † | † | † | † | † |

ICC, intraclass correlation coefficient; † No data available for assessment
