# Supplementary material for: Evaluation of psychometric properties of needs assessment tools in cancer patients: A systematic literature review
Source: PLoS One. 2019 Jan 8;14(1):e0210242. doi: 10.1371/journal.pone.0210242 (PMC6324833; doi:10.1371/journal.pone.0210242)
Supplement: S4 Table — (DOCX) [file pone.0210242.s007.docx]

**S4 Table. Validity assessment of needs assessment tools in cancer patients**

| Instrument | Language | Content validity | Construct validity | | Cross-cultural validity |
| --- | --- | --- | --- | --- | --- |
|  |  |  | Structural validity | Hypothesis testing |  |
| SCNS-SF | English [13] | Selecting items based on clinical relevance and statistical evidence | Confirmatory factor analysis, 5 factors explaining 73.0% of the variable | 1. Convergent validity, correlations with HADS, DT and QLQ-C30  2. Discriminant validity, scores differentiated in patients with or without remission | † |
|  | French [46] | 1. Translation of original scale  2. Pilot testing | Confirmatory factor analysis, 5 factors allowing correlations between residuals of 12 redundant pairs of items (RMSEA=0.05; CFI=0.98; TLI-NNFI=0.98) | 1. Convergent validity, correlations with QLQ-C30  2. Discriminant validity, score discriminated between patients according to stage of disease, age, having children or not, and education level | 1. Forward-backward translation  2. Pilot study |
|  | German [22] | Translation of original scale | Exploratory factor analysis, 5 factors explaining 68.0% of the variable | 1. Convergent validity, moderate correlations between psychological domain and HADS; low association between each domain, and DT, HADS and detrimental interaction  2. Discriminant validity, significant differences between patients with respect to sex and age | Forward-backward translation |
|  | Japanese [47] | Translation of original scale | Exploratory factor analysis, 5 factors explaining 74.6% of the variance | 1. Convergent validity, correlation with QLQ-C30  2. Discriminant validity, scores differentiated between patients according to physical condition, and age | Forward-backward translation |
|  | Traditional Chinese [23] | Mandarin and Cantonese SCNS-SF was used [27] | Confirmatory factor analysis supported 5 factors in Hong Kong and Taiwan samples allowing correlations between residuals of some items | 1. Convergent validity, correlations with PSQ-9 satisfaction  2. Discriminative validity, significant differences emerged according to age | † |
|  | Mandarin [24] | 1. Translation of original scale  2. Pilot testing | Exploratory factor analysis, 5 factors explained 69.8% of the variance | 1. Convergent validity, moderate correlations with HADS and QLQ-C30, and weak correlations with SSRS  2. Discriminant validity, significant differences between patients with respect to disease stage, sex and age | 1. Forward-backward translation  2. Pilot testing |
|  | Mandarin and Cantonese [48] | Translation of original scale | Exploratory factor analysis, 4 factors explained 54.2% of the variance | 1. Convergent validity, moderate correlations with HADS, CHQ-12, MSAS, and PANAS  2. Discriminant validity, significant differences between patients with respect to treatment status, disease stage and age | Forward-backward translation |
|  | Spanish [25] | 1. Translation of original scale  2. Expert review | Exploratory factor analysis, 5 factors explained 59.0% of the variance | 1. Convergent validity, correlation with HADS and QLQ-C30  2. Discriminative validity, significant differences between patients with respect to disease stage, treatment, sex and age | Forward-backward translation |
|  | Dutch [49] | 1. Translation of original scale  2. Examining redundancy and completeness of items | Confirmatory factor analysis, previous factor structure cannot be replicated  Exploratory factor analysis, 4 factors explaining 50.6% of the variance | 1. Construct validity, 96% of hypothesised correlations between SCNS-SF and other PROMs were found, and 57% supported a priori hypothesised magnitude of correlation  2. Discriminant validity, significant differences between patients with regard to treatment and treatment period | Forward-backward translation |
|  | English [50] | † | Exploratory factor analysis, 5 factors | 1. Convergent validity, correlations with HADS, and EPIC hormonal summary  2. Divergent validity, low correlations with EPIC domains (urinary, bowel and sexuality) | † |
| SCNS-ST9 | English [26] | 1. Diagnostic accuracy (sensitivity and specificity)  2. Selecting items based on variance, factor loading and item prevalence  3. Predictive ability | † | † | † |
| SCNAT-IP | English [27] | 1. Qualitative study on patients  2. New Indigenous-specific items were added | Exploratory factor analysis, 4 factors explaining 50.9% of the variance | 1. Construct validity, correlated with DT, CWC, and AQoL-4D  2. Discriminant validity, unmet needs scores differed according to sex, age, settings, education level and regions | † |
| CANDI | English [16] | 1. Items came from patients  2. Items were revised based on theoretical model and literature  3. Expert and patients review | † | 1. Convergent validity, correlation with HADS and BSI  2. Discriminant validity, depression subscale discriminated between depressed and non-depressed patients, and between anxious and non-anxious patients | † |
|  | Turkish [28] | 1. Translation of original scale  2. Pilot study | Exploratory factor analysis, 3 factors | 1. Convergent validity, correlations with HADS and BSI  2. Divergent validity, moderate and significant correlations between CANDI and FACT-G | 1. Forward-backward translation  2. Pilot testing |
| CARES-SF | English [31] | Expert review | Confirmatory factor analysis, 6 summary scales | Convergent validity, correlations with QOL, SCL-90 and KPS | † |
| CARES | English [29] | 1. Patients and experts review  2. Post-test patient interview | † | Convergent validity, correlations with SCL-90 and KPS | † |
|  | Flemish [30] | Translation of original scale | Exploratory factor analysis, 5 summary scales explaining 65.5% of the variance | Concurrent validity, correlation with KPS, HADS, D-subscale of SSL, MMQ-M and MMQ-S, QLQ-C30 and DT | Forward-backward translation |
| CaSUN | English [14] | 1. Literature review  2. Qualitative studies on patients  3. Expert review | Exploratory factor analysis, 5 sections explaining 54.0% of the variance | Concurrent validity, correlation with age, number of treatments, mental QOL, physical QOL, anxiety and depression | † |
| CaSUN-NL | Dutch [32] | 1. Translation of original scale  2. Addition of lifestyle and return to work subscale  3. Pilot study | Exploratory factor analysis, 6 sections explaining 51.0% of the variance | Construct validity, correlation with age, marital status, anxiety, depression, positive and negative adjustment, and global health | 1. Forward-backward translation  2. Pilot testing |
| CaSUN-C | Chinese [51] | 1. Translation of original scale  2. Addition of breast-specific subscale | Exploratory factor analysis, 4 sections (sample 1)  Confirmatory factor analysis demonstrated 4 factors (sample 2) | 1.Concurrent validity, correlation with CES-D and fear of recurrence  2. Discriminant validity, scores differentiated according to gender, age, and time for treatment | Forward-backward translation |
| SUNS | English [15] | 1. Literature review  2. Patient and expert consultation  3. Pilot testing | Exploratory factor analysis, 5 subscales explaining 64.4% of the variance | † | † |
|  | English [52] | 1. Experts review  2. Patient interview | Exploratory factor analysis, 5 subscales | 1. Convergent validity, correlation with DASS-21  2. Discriminant validity, scores differentiated according to recurrence, age, and treatment | † |
| SUNS_ SF | English [33] | 1. Researchers discussion  2. Items removal using theoretical and statistical methods | Exploratory factor analysis, 4 subscales | Discriminant validity, scores differentiated according to treatment | † |
| SPARC | English [54] | 1. Literature review  2. Patient and professional interview  3. Cognitive interviewing  4. Expert consultation  5. Pilot study | † | † | † |
|  | Polish [34] | 1. Translation of original scale  2. Pilot testing | Exploratory factor analysis, 6 subscales | Discriminant validity, significant difference in religious and spiritual issues and independence and activity domains between patients in day care, home care and palliative care | 1. Forward-backward of translation  2. Pilot testing |
| NA-ACP | English [35] | 1. Literature review  2. Professional and patient consultation  3. Pilot testing | Exploratory factor analysis, 7 subscales explaining 55.0% of the variance | † | † |
| NA-ALCP | English [53] | 1. Expert review  2. Pilot testing | † | Convergent validity, correlations with QLQ-C30, HADS and DT | † |
| SPEED | English [36] | Experts consultation | † | † | † |
| 3LNQ | Danish [37] | Literature review | † | † | † |
| CNAT | Korean [38] | 1. Reviewed needs tools  2. Qualitative study on patients  3. Expert and patient consultation  4. Pilot testing | Exploratory factor analysis, 7 factors explaining 64.2% of variance | 1. Convergent validity, correlation with EQ5D  2. Discriminant validity, significant differences with regard to sex, education, insurance, cancer type, stage, treatment | † |
| CNQ-SF | English [39] | † | Exploratory factor analysis, 5 factors explaining 68.0% of variance | 1. Convergent validity, correlation with QLQ-C30 and BDI-SF  2. Discriminant validity, significant differences according to disease state, gender, performance status, treatment, fatigue | † |
| PNPC | Dutch [40] | 1. Qualitative study on cancer patients and professionals  2. Literature review  3. Expert consultation | † | Convergent validity, correlation with HRQOL dimensions | † |
| ISQ | Greek [41] | Translation of original version | Exploratory factor analysis, 2 subscales explaining 70.6% of the variance | Discriminant validity, significant differences according to shared decision making with their doctor, age, diagnosis, educational level and the existence of metastasis | Forward-backward translation |
| SST-IUPCN | English [42] | Experts consultation | † | 1. Concurrent validity, patients positive for uncontrolled symptoms had a higher pain intensity and physical symptom distress  2. Predictive validity, patients in need of a consult had higher score  3. Discriminant validity, significant difference based on survival period | † |
| NEQ | Italian [43] | 1. Patient interview  2. Pilot study | Confirmatory factor analysis demonstrated 4 factors | † | † |
|  | Italian [44] | † | Exploratory factor analysis in subsample 1, 5 factors explaining 50.5% of the variance  Confirmatory factor analysis demonstrated 5 factors in subsample 2 with NNFI=0.97; CFI=0.98; RMSEA=0.03 | † | † |
|  | Italian [45] | † | Confirmatory factor analysis extracted 5 factors with TLI=0.98; CFI=0.96; RMSEA=0.05 | Discriminant validity, significant differences between inpatients and outpatients | † |

† No data available for assessment

HADS, Hospital anxiety and depression scale, EORTC QLQ-C30, The 30-item European organization for research and treatment of cancer quality of life core questionnaire, EORTC QLQ-INFO25, The 25-item EORTC QLQ information module, SSRS, Social support rating scale, BSI, Brief symptom inventory, KPS, Karnofsky performance status scale, SSL-I and-D, Social support list-interactions and discrepancies, MMQ, Maudsley marital questionnaire, DT, Distress thermometer, CWC, Cancer worry chart, AQoL-4D, Assessment of quality of life-4D, DASS-21, Depression anxiety and stress scale-21, PSQ-9, Patient satisfaction questionnaire-9, BDI-SF, Beck depression inventory short-form, SCL-90, Symptom checklist-90, EPIC, Expanded prostate cancer index composite-short form, CHQ12, Chinese health questionnaire, MSAS, Memorial symptom assessment scale, PANAS, Positive and negative affect schedule
